# CAREGIVING INTERACTIONS OF FORMAL CAREGIVERS AND PERSONS WITH DEMENTIA: A SYSTEMATIC REVIEW OF MEASURES

**DOI:** 10.1093/geroni/igad104.0814

**Published:** 2023-12-21

**Authors:** Anju Paudel, Wen Liu, Ying-Ling Jao, Thakshila Dasanayake, Yo-Jen Liao, Nahida Akter

**Affiliations:** Penn State Ross and Carol Nese College of Nursing, State College, Pennsylvania, United States; The University Of Iowa, Iowa City, Iowa, United States; Pennsylvania State University, State College, Pennsylvania, United States; The Pennsylvania State University, State College, Pennsylvania, United States; Penn State University, State College, Pennsylvania, United States; Penn State University, State College, Pennsylvania, United States

## Abstract

A psychometrically sound assessment of caregiving interactions is essential to collect valid information about the interactions, and to establish interventions to optimize the interactions in dementia care. This review aims to identify measures of caregiving interactions between formal caregivers and persons with dementia and evaluate their psychometric properties. We conducted a systematic review of studies published by February 2023 in four databases— PubMed, PsychINFO, CINHAL, and Web of Science. COnsensus-based Standards for the selection of health status Measurement INstruments (COSMIN) checklist was chosen for quality appraisal of selected studies. Our search yielded 818 scholarly records and identified five eligible instruments (published in six scholarly records). Three measures assessed interactions between formal caregivers and persons with dementia from both caregiver and person with dementia’s perspectives while one evaluated from the caregiver perspective and the other from person with dementia’s perspective. Additionally, three measures assessed interactions based on observations while other two were based on interviews. Preliminary results showed the Communication Behavior in Dementia (CODEM) as a promising measure with some excellent psychometrics but it needed further testing with larger sample and assessment of responsiveness to change. The remaining four measures (IC-Communication-SR, Coding Scheme for Mealtime Interactions, Montreal Evaluation of Communication Questionnaire for use in Long-Term Care (MECQ-LTC), and Barnards Feeding Scale) had concerns related to low internal consistency, low reliability, and lack of adequate psychometric tests. Overall, this review identified gaps in current measures, suggesting a need to establish psychometrically sound tools to assess and optimize caregiving interactions in dementia care.

